# Characterization of Adherent Bacteroidales from Intestinal Biopsies of Children and Young Adults with Inflammatory Bowel Disease

**DOI:** 10.1371/journal.pone.0063686

**Published:** 2013-06-11

**Authors:** Naamah L. Zitomersky, Benjamin J. Atkinson, Sarah W. Franklin, Paul D. Mitchell, Scott B. Snapper, Laurie E. Comstock, Athos Bousvaros

**Affiliations:** 1 Division of Gastroenterology, Boston Children's Hospital, Harvard Medical School, Boston, Massachusetts, United States of America; 2 Clinical Research Center, Boston Children's Hospital, Boston, Massachusetts, United States of America; 3 Division of Gastroenterology, Brigham & Women's Hospital, Boston, Massachusetts, United States of America; 4 Division of Infectious Diseases, Brigham and Women's Hospital, Harvard Medical School, Boston, Massachusetts, United States of America; Instutite of Agrochemistry and Food Technology, Spain

## Abstract

There is extensive evidence implicating the intestinal microbiota in inflammatory bowel disease [IBD], but no microbial agent has been identified as a sole causative agent. Bacteroidales are numerically dominant intestinal organisms that associate with the mucosal surface and have properties that both positively and negatively affect the host. To determine precise numbers and species of Bacteroidales adherent to the mucosal surface in IBD patients, we performed a comprehensive culture based analysis of intestinal biopsies from pediatric Crohn's disease [CD], ulcerative colitis [UC], and control subjects. We obtained biopsies from 94 patients and used multiplex PCR or 16S rDNA sequencing of Bacteroidales isolates for species identification. Eighteen different Bacteroidales species were identified in the study group, with up to ten different species per biopsy, a number higher than demonstrated using 16S rRNA gene sequencing methods. Species diversity was decreased in IBD compared to controls and with increasingly inflamed tissue. There were significant differences in predominant Bacteroidales species between biopsies from the three groups and from inflamed and uninflamed sites. *Parabacteroides distasonis* significantly decreased in inflamed tissue. All 373 Bacteroidales isolates collected in this study grew with mucin as the only utilizable carbon source suggesting this is a non-pathogenic feature of this bacterial order. *Bacteroides fragilis* isolates with the enterotoxin gene [*bft*], previously associated with flares of colitis, were not found more often at inflamed colonic sites or within IBD subjects. *B. fragilis* isolates with the ability to synthesize the immunomodulatory polysaccharide A [PSA], previously shown to be protective in murine models of colitis, were not detected more often from healthy versus inflamed tissue.

## Introduction

Inflammatory bowel diseases, which include Crohn's disease [CD] and ulcerative colitis [UC], are chronic, inflammatory, disorders of the gastrointestinal tract defined by clinical, pathological, endoscopic, and radiologic features [1]. The precise etiology of IBD is unknown. The current predominating hypothesis is that intestinal inflammation in IBD arises from an exaggerated immune response to the intestinal microbiota in genetically predisposed individuals. A role for intestinal bacteria in the pathogenesis of IBD is supported by clinical data. Patients who undergo surgical diversion of the fecal stream revert to uninflamed healthy intestines. However, when this previously excluded intestinal epithelium is re-exposed to the microbial laden fecal stream, inflammation recurs [2]. In addition, antibiotics that target anaerobic gut bacteria have shown some efficacy in the treatment of IBD [3], [4].

Multiple studies have shown that the most numerically dominant bacteria in the human gut belong to two phyla: the Gram negative Bacteroidetes and the Gram positive Firmicutes [5]. The human gut Bacteroidetes are largely contained within the order Bacteroidales, which includes over 20 abundant human gut species. As fecal/luminal bacterial communities differ from mucosal communities [6], and the site of inflammation in IBD occurs at the mucosal surface, several studies have analyzed the mucosal associated bacterial population in IBD [7], [8], [9]. No clear consensus has been reached about total Bacteroidales levels or Bacteroidales species distribution between IBD subjects and controls, or between CD and UC [7], [9]. However, Bacteroidales have been consistently shown to predominate at the mucosal surface where they are more transcriptionally active than Firmicutes [10].

Because Bacteroidales occupy a vital niche at the intestinal mucosal surface, they are able to interact with the host and have been shown to modulate host immune and intestinal functions including mucosal barrier fortification [11], intestinal immune maturation [12], and angiogenesis [13]. Bacteroidales species can confer either beneficial or detrimental properties to the host depending upon their genetic content. *Bacteroides fragilis* type strain NCTC9343 synthesizes a polysaccharide [PSA] demonstrated to suppress colitis in an experimental animal model [12], [14]. However, only 26% of the *B. fragilis* strains analyzed were shown to synthesize the same PSA molecule as the type strain [15] and therefore, not all *B. fragilis* strains may have this immunomodulatory capability. In addition, some *B. fragilis* strains produce an enterotoxin which induces colitis and colon cancer in animal models and has been associated with colitis flares in human studies [16]. Due to the extensive genetic and phenotypic variability of these organisms, geno- and phenotyping of *B. fragilis* strains from IBD patients is important to determine if there is a correlation between the presence *B. fragilis* strains synthesizing these molecules at the mucosal surface, and the progression or amelioration of disease.

Mouse models of IBD have shown that Bacteroidales species interact differently with the host depending on the immunological defect and the particular bacterial species used [reviewed [17]]. For example, gnotobiotic -HLA-B27 transgenic rats develop colitis when monoassociated with *B. vulgatus*, but not with *Escherichia coli* or *Enterococcus faecalis* [18]. However, gnotobiotic IL-10 KO mice monoassociated with *B. vulgatus* develop colitis but not with *E. coli* or *E. faecalis* [19]. Conventionally housed mice, with a double knock out of the IL-10 receptor 2 and a dominant negative TGF-β [a combination of pathways often defective in subjects with IBD], developed intestinal inflammation only in the presence of certain Bacteroidales species [20]. Furthermore, treatment with antibiotics targeting Bacteroidales ameliorated disease [20]. This differential effect of *Bacteroides* species in IBD mouse models suggests the importance of characterizing Bacteroidales species distribution at the gut mucosal surface of IBD patients.

Pediatric IBD is increasing in incidence and in younger patients [21]. Pediatric patients often have a more aggressive disease and a more rapid progression. However, only two prior studies have examined intestinal biopsy tissue from pediatric subjects with IBD [22] [23]. These studies had fewer patients and biopsies, and did not speciate or phenotypically characterize the resulting strains. Our study is the largest study using biopsy tissue from pediatric and young adult subjects with IBD including both treatment naive patients and those with pre-existing IBD. In this study, we performed a comprehensive culture-based analysis of Bacteroidales from mucosal biopsy tissue from pediatric subjects with CD, UC, and control subjects.

This study addresses several objectives. Using culture based methods, we quantified total mucosal Bacteroidales from IBD and control subjects to determine if the levels increased or decreased with the inflammatory state. We characterized which species of Bacteroidales are present at inflamed and non-inflamed mucosal sites in IBD subjects and in healthy control subjects and found previously unreported differences in Bacteroidales species distribution. We tested Bacteroidales isolates for strain specific features previously postulated to be protective or pathogenic in IBD. We assessed pro-inflammatory factors such as toxin production by *B. fragilis*, and the ability of Bacteroidales to use mucin as a carbon source, both features associated with diminishing host immune defenses [16], [24]. We also assessed production of immunomodulatory PSA shown to be protective in murine colitis models. This study is the first to contain phenotypic analyses of the adherent Bacteroidales population from pediatric IBD subjects.

## Materials and Methods

### Patients and acquisition of samples

This study was approved by the Institutional Review Board of Boston Children's Hospital and informed written consent was obtained from study subjects and/or their legal guardians. Subjects with previously diagnosed or suspected IBD undergoing colonoscopy were recruited as well as control subjects without IBD or other intestinal inflammatory disease. Subjects were recruited at Boston Children's Hospital Gastroenterology endoscopy unit. 39 subjects had CD, 24 subjects had UC, and there were 31 control subjects (Table 1). Twelve subjects with CD and five subjects with UC were newly diagnosed. The majority (84%) of our subjects had three biopsies taken (4 subjects had only 2 biopsies, and the first 6 subjects had six biopsies) with standard forceps from the terminal ileum, cecum to descending, and recto-sigmoid colon from grossly inflamed (when present) and non-inflamed sites [Table S1]. The presence or absence of inflammation was determined by histopathological review revealing that 79% of subjects with IBD had *any* degree of inflammation, and 41% were classified as moderate to severe inflammation. Control subjects did not have histologic evidence of intestinal inflammation detected on any of their biopsies. Inflammation status and sample location are indicated in Table S1. There were no significant differences in mean age (*P* = 0.22) or gender (*P* = 0.45) between control, CD, or UC cohorts. Disease phenotyping followed the Montreal classification [25] and disease activity was assessed using the Pediatric Ulcerative Colitis Activity Index (PUCAI) [26] and Pediatric Crohn's Disease Activity Index (PCDAI) [27]. Other documented clinical parameters included medications, prior surgery, and disease activity. Control subjects with esophagitis or gastritis had no detected granulomas. Control subjects were ultimately diagnosed with one or more the following: gastroesophageal reflux [9], functional abdominal pain [6], constipation [4], irritable bowel syndrome [5], isolated rectal bleeding [2], *Helicobacter pylori* gastritis [2], non-specific severe gastritis [1], Meckel's diverticulum [1], and infectious diarrhea [1]. Some control subjects had autoimmune disorders; subject 8 has rheumatoid arthritis, subject 19 has cutaneous plaque morphia, subject 30 has psoriasis and psoriatic arthritis, subject 50 has type 1 diabetes, and subject 96 has multiple sclerosis.

**Table 1:** Study population demographics and disease phenotype.

		Control (*n* = 31)	Crohn Disease (*n* = 39)	Ulcerative Colitis (*n* = 24)
**Age** (years)	Mean ± SD (*P = *0.22)	14.6±3.8	15.3±4.6	16.6±4.5
	Range	7.1–22.9	5.1–23.4	3.7–23.6
**Female** (*P* = 0.45)		19	18	13
**Immunosuppressed**		1	21	8
**Montreal criteria**	Ileal disease (L1)		10	
	Colonic disease (L2)		3	
	Ileocolonic disease (L3)		26	
	Isolated upper tract disease		0	
	Non-Stricturing/Penetrating (B1)		29	
	Stricturing/Penetrating (B2/B3)		9	
	Perianal Disease (P)		16	
**Ulcerative colitis type**	Pancolitis (E1)			11
	Left sided colitis (E2)			11
	Proctitis <15cm (E3)			2

1. *n* = subject.

2. Immunosuppressed is defined as any patients taking immunomodulators and/or biologics.

### Biopsy Processing and Culture

Each biopsy was placed in sterile 0.9% NaCl [saline] and transported to the laboratory at room temperature for immediate processing. Biopsies were washed three times by vigorously shaking with saline to remove non-adherent bacteria. Biopsies were then homogenized using a manual pestle in an eppendorf tube. Samples were diluted in PBS and plated on Brucella laked blood, kanamycin, vancomycin (LKV) plates (PML Microbiologicals, Durham, NC, USA), which are selective for Bacteroidales. All species of *Bacteroides*, *Parabacteroides*, and *Prevotella* tested grew on these plates. *Alistipes* species were excluded from this study as it was unclear whether these organisms grow under these culture conditions. The plates were placed in an anaerobic chamber at 37°C for 4 days. Dilution plates with between 20–200 colonies were recorded [usually the 10^−4^, 10^−3^ or 10^−2^ plates] and the species were identified by a series of multiplex PCR assays (Table S2). If colonies were only detected on the 10^−1^ plate, these numbers were recorded. All colonies on the highest dilution plate were identified at the species level and several isolates representing different colony phenotypes (large, small, mucoid, color) on the second highest dilution plate were identified. The number of colonies analyzed ranged from 0–73 (mean 34.5±14.5 standard deviation (SD)) per subject and 0–31 (mean 11.2±6.1 SD) per biopsy (depending on colony phenotypes). One isolate of each species from an inflamed and non-inflamed site identified at each time point was frozen at −80°C.

### Multiplex PCR assays

**T**he multiplex PCR assays are based on the design of Liu et. al. [28] and take advantage of the variable region between the 16S and 23S rRNA genes. All primers used in this study are listed in Table S2. Taq Mastermix (New England Biolabs Inc. Ipswich, MA, USA) was used for all PCRs and the conditions were as follows: multiplex I, 2 minutes 94°C, then 35 cycles of 94°C 30 s, 59°C 30 s, 68°C 45 s with a final 60 s extension at 68 °C. The same conditions were used for multiplex II except the annealing temperature was 52°C. PCR products were resolved on 1.4% agarose gels. Larger colonies were first screened using multiplex I, if the species was not identified, multiplex II was performed. Small colonies were first screened using multiplex II, followed by multiplex I if they were negative with multiplex II. In total, 1450 isolates were speciated using this multiplex PCR. Additional primers were used to differentiate species that showed inconclusive results as previously described [42]. 134 isolates were analyzed to differentiate *B. vulgatus* from *B. dorei*; 61 isolates were screened to differentiate *B. ovatus* from *B. xylanisolvens* and *B. acidifaciens*; and 34 strains were screened to differentiate *B. fragilis* from *B. finegoldii*. All isolates identified as *B. stercoris* by multiplex PCR were further analyzed by sequencing the 286 bp 16S gene region. Isolates unresolved by multiplex PCR, or the above primer sets, were speciated by sequencing a 286 bp region of the 16S gene region or the full 16S gene (Table S2). In total 120 isolates had the 286 bp region sequenced, and full 16S sequencing was performed for an additional 26 isolates.

### PCR screening of *B. fragilis* isolates for the enterotoxin gene [*bft*]

PCR was performed using previously described primers (Table S2), which amplify all three isoforms of *bft* [29] with the following PCR conditions: 95°C for 2 min and then 32 cycles of 95°C for 30 s, 62°C for 30 s, and 68°C for 60 s. In total, 104 *B. fragilis* isolates were screened for the presence of *bft*.

### *B. fragilis* polysaccharide A [PSA] dot blot

Three μl of *B. fragilis* grown to stationary phase were dotted on nitrocellulose membranes. The primary antibody was monoclonal antibody CE3 to the PSA of type strain *B. fragilis* NCTC 9343 [15]. The secondary antibody was an alkaline phosphatase-conjugated goat-anti-mouse IgG (Sigma-Aldrich, St. Louis, MO, USA). Blots were developed with phosphatase substrate (KPL, Gaithersburg, MD, USA). Any isolate that produced an ambiguous result was retested by Western immunoblot with the same primary and secondary antibodies. In total, all 113 isolates of *B. fragilis* that were retained as frozen stocks were analyzed.

### Growth of strains on defined mucin agarose plates

Defined medium agarose plates [30] lacking glucose and containing 0.3% porcine gastric mucin (Sigma-Aldrich, St. Louis, MO, USA) as the sole utilizable carbon source were inoculated with strains and incubated anaerobically for 96 hours, at which time growth was monitored. Fourteen Bacteroidales species were tested on these defined agarose plates [30] lacking mucin and none grew.

### Statistical Analysis

Study data were collected and managed using REDCap electronic data capture tools hosted at Boston Children's Hospital [31]. REDCap (Research Electronic Data Capture) is a secure, web-based application designed to support data capture for research studies, providing [1] an intuitive interface for validated data entry [2] audit trails for tracking data manipulation and export procedures [3] automated export procedures for seamless data downloads to common statistical packages, and [4] procedures for importing data from external sources. Statistical analysis was conducted in SAS version 9.2 (SAS Institute, Cary, NC) and graphics created with SAS and Microsoft Excel (Microsoft Excel. Redmond, Washington: Microsoft, 2003, Computer Software).

Categorical data were summarized as N (%) and comparisons across groups made by the Pearson Chi-Square statistic, or Fisher's exact test when the expected cell count was <5 in one or more cross-tabulation table cells. Continuous variables were summarized according to their distribution: mean ± SD if normally distributed; median (inter-quartile range) if skewed right or left. The method for group comparisons for continuous variables was likewise chosen to suit the distribution of outcome. Student's t-test was used to compare normally distributed variables between two independent groups, and analysis of variance in the case of three or more independent groups. Non-parametric equivalents (Wilcoxon rank-sum test; Kruskal-Wallis test) were used otherwise. Generalized estimating equations [GEE] were used to compare means across groups, controlling for within-subject correlation. All tests of significance are 2-sided with P<0.05 indicating statistical significance. The correlation of total Bacteroidales per biopsy with number of species identified per biopsy was examined using Spearman rank correlation.

## Results

### Total Bacteroidales per biopsy by cohort and degree of inflammation

**Figure 1:**
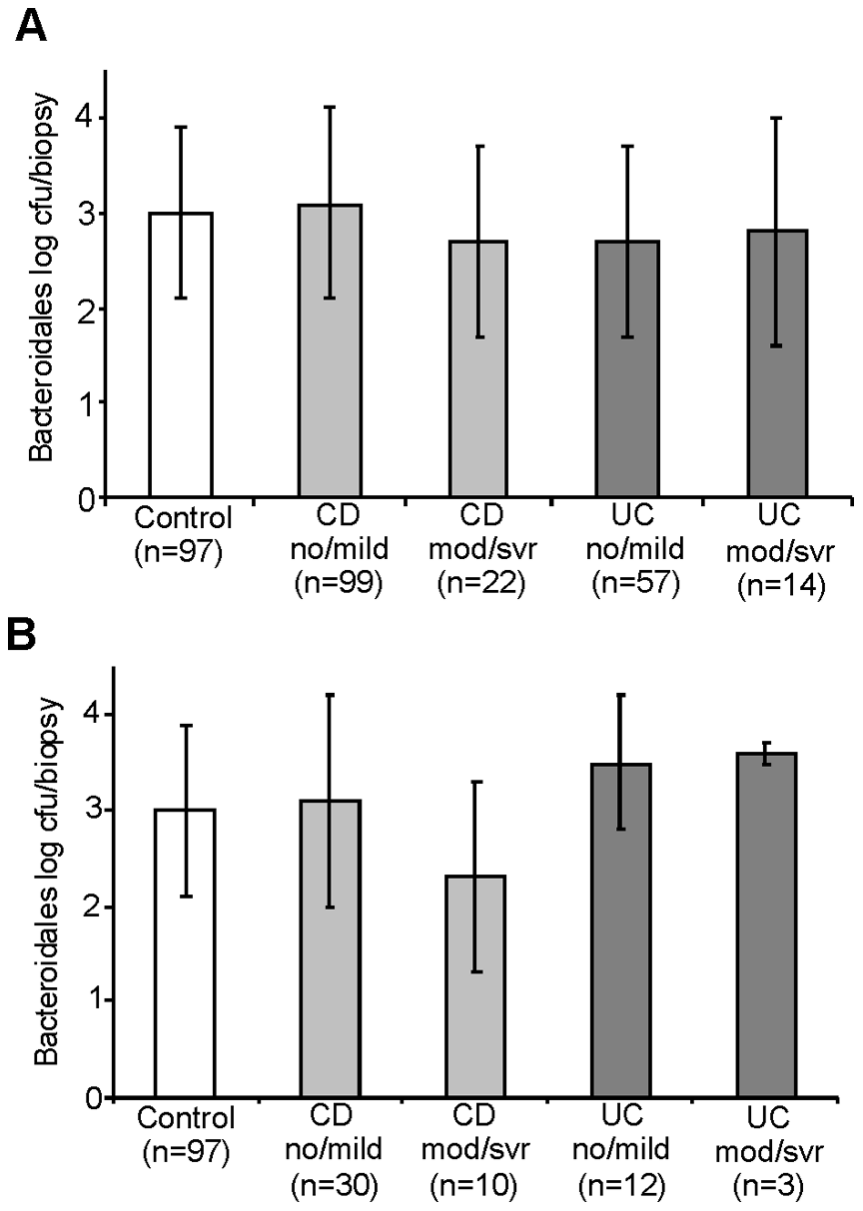
Total Bacteroidales (cfu/biopsy) by cohort and degree of inflammation. Biopsies were grouped by cohort: CD (Crohn Disease), UC (ulcerative colitis) and Control and degree of inflammation: no and mild inflammation (no/mild) or moderate to severe inflammation (mod/svr). N is equivalent to the number of biopsies in each group. Column level represents mean Bacteroidales log cfu/biopsy. Error bars represent inter-quartile ranges **A.** Entire cohort. There were no significant differences detected between cohorts. **B** Includes only biopsies from newly diagnosed subjects.

Because some studies have shown that Bacteroidales differ in quantity at inflamed sites, we analyzed IBD cohorts by degree of inflammation combining moderate to severely inflamed biopsies, and biopsies with no or mild inflammation. Total adherent Bacteroidales ranged from zero to 4 log colony forming units (cfu) per biopsy. Total Bacteroidales per biopsy did not differ significantly based on biopsy location from terminal ileum to rectum (data not shown). Control biopsies had a mean ± SD of 3.0±0.9 cfu/biopsy which was not statistically different than the combined IBD cohort mean ± SD of 2.9±1.0 cfu/biopsy *P* = 0.74 (Table 2). When examining the entire IBD cohort, total Bacteroidales among inflamed biopsies were similar mean ± SD 2.8±1.1 to uninflamed IBD biopsies 3.0±1.0 *P* = 0.28.(Figure 1A, Table 2). When examined by severity of inflammation or comparing UC and CD, no significant differences in total Bacteroidales per biopsy were detected. Analysis of treatment naive subjects showed no significant differences in total Bacteroidales per biopsy by cohort or by degree of inflammation (Figure 1B, Table S3).

**Table 2:** Total Bacteroidales [cfu/biopsy) and number of different Bacteroidales species detected per biopsy by diagnoses and degree of inflammation in entire cohort.

		Subjects	Biopsies	Total Bacteroidales cfu/biopsy (mean log cfu/biopsy ± SD)	*P*	Bacteroidales species identified per biopsy Median (IQR)	*P*
**Group (all)**	IBD	63	192	2.9±1.0	0.74	3 (1, 4)	0.09
	Control	31	97	3.0±0.9		3 (2, 4)	
	CD	39	121	3.1±1.0	0.19	3 (1, 4)	0.95
	UC	24	71	2.7±1.1		3 (1, 4)	
	CD	39	121	3.1±1.0	0.75	3 (1, 4)	0.13
	Control	31	97	3.0±0.9		3 (2, 4)	
	UC	24	71	2.7±1.1	0.28	3 (1, 4)	0.24
	Control	31	97	3.0±0.9		3 (2, 4)	
**IBD (63 subjects)**	Inflammation	50	87	2.8±1.1	0.28	3 (1, 4)	0.38
	No Inflammation	56	105	3.0±1.0		2.5 (2, 4)	
	Moderate/severe inflammation	26	36	2.7±1.1	0.22	2 (1, 3)	**0.03**
	No/mild Inflammation	62	156	3.0±1.0		3 (2, 4)	
**CD (39 subjects)**	Inflammation	27	47	2.9±1.0	0.33	2 (1, 3)	0.63
	No inflammation	34	74	3.1±1.0		3 (2, 4)	
	Moderate/severe inflammation	16	22	2.7±1.0	0.12	2.5 (1, 3)	0.09
	No/mild Inflammation	38	99	3.1±1.0		3 (2, 4)	
**UC (24 subjects)**	Inflammation	23	40	2.7±1.1	0.98	3 (1, 4)	0.41
	No inflammation	22	31	2.7±1.0		2 (2, 4)	
	Moderate/severe inflammation	10	14	2.8±1.2	0.85	1.5 (0, 3)	0.16
	No/mild Inflammation	24	57	2.7±1.0		3 (2, 4)	

*P*-value from generalized estimating equation, controlling for within-subject correlation.

All but two control subjects (4, 50) had at least 10 cfu/biopsy, and usually more, whereas five UC subjects (9, 18, 55, 90, and 99) and six CD subjects (17, 27, 54, 65, 70, and 76) had no Bacteroidales detected on one or more of their biopsies. Of these five UC subjects, one (99) had no Bacteroidales detected on any biopsies and one (18) had Bacteroidales only detected on one biopsy at a low level (10 cfu/biopsy). Of the six with CD, one subject (76) had no Bacteroidales detected on any biopsies, and five subjects (17, 27, 54, 65, and 70) had none detected on just one biopsy. When subtracting the two newly diagnosed subjects with CD from the group, seven of eight had one characteristic in common, they were on a 5-aminosalicylic acid medication (5-ASA) alone or in combination with other anti-inflammatory medications.

### Species Identification and Diversity

In total, from all subjects and sites, 18 different Bacteroidales species were identified: 14 *Bacteroides* species, two *Parabacteroides* species, a *Dysgonomonas gadei* and *Odoribacter splanchnicus* [Porphyromonadaceae family] and a *Prevotella bivia* [Prevotellaceae family]. Infrequently, non-Bacteroidales species that had acquired antibiotic resistance allowing them to grow on these selective plates were detected. These species were *Clostridium innocuum, Pediococcus acidilactici, E. coli, Staphylococcus epidermidis, Propionibacterium acnes, Proteus sp*., and *Staphylococcus sp*. There was a positive correlation of 0.47 (*P*<0.0001) between total Bacteroidales concentration per biopsy with the number of species identified per biopsy, by Spearman rank correlation.

**Figure 2:**
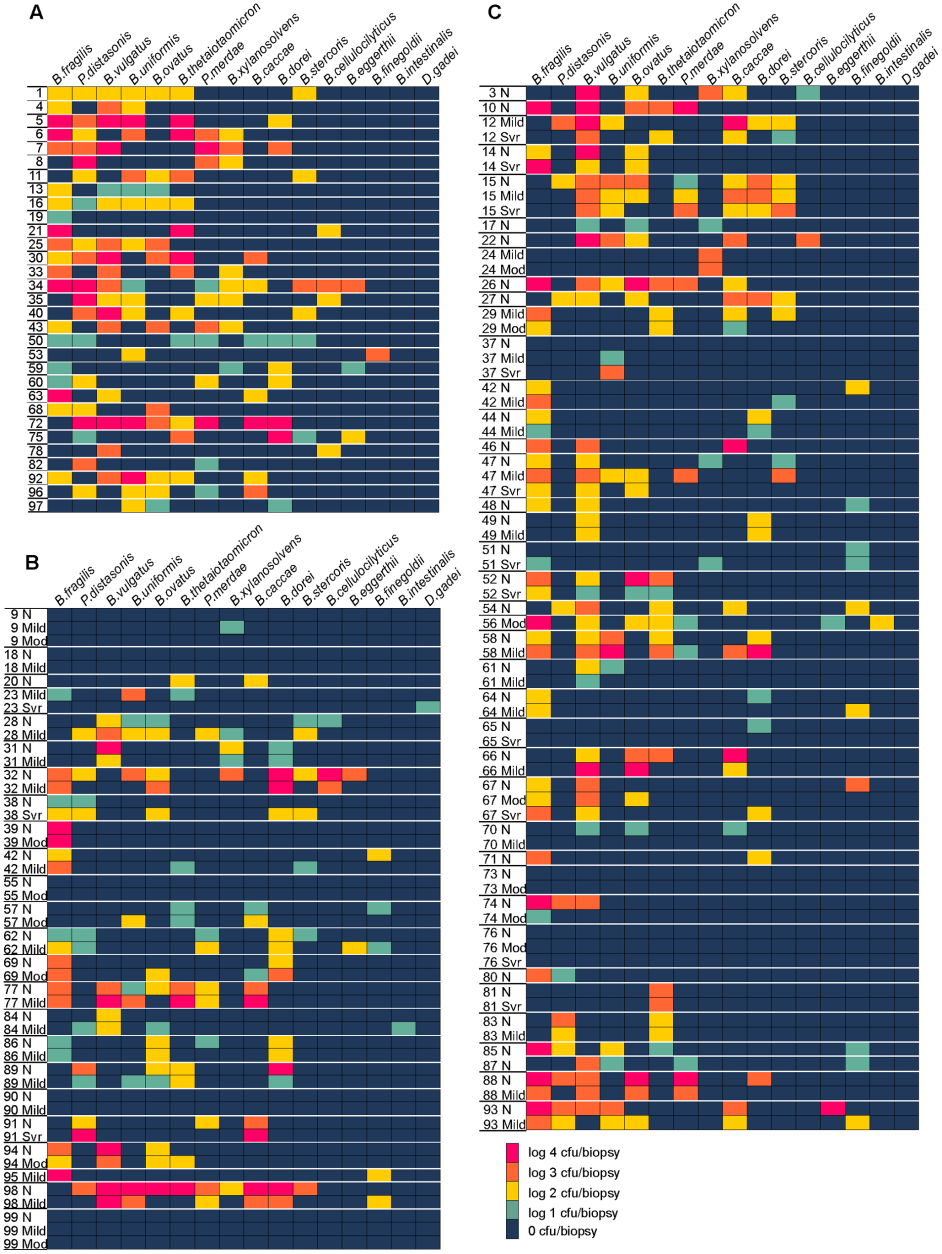
Heatmaps of Bacteroidales species distribution and concentration by cohort. **A.** Control cohort. **B.** UC cohort. **C.** CD cohort. Bacteroidales species are labeled at the top of the figure. Subject numbers are listed on the left. Each row represents the species detected on that subject's biopsy (In cases where more than one biopsy of the same degree of inflammation was present, the detected species levels (cfu/biopsy) from each biopsy was averaged and all detected species from both biopsies were included). The degree of inflammation of the biopsy is indicated to the right of the biopsy (N: Non-inflamed, Mild, Mod: Moderate, or Svr: Severe). White lines demarcate biopsies from one subject.

**Figure 3:**
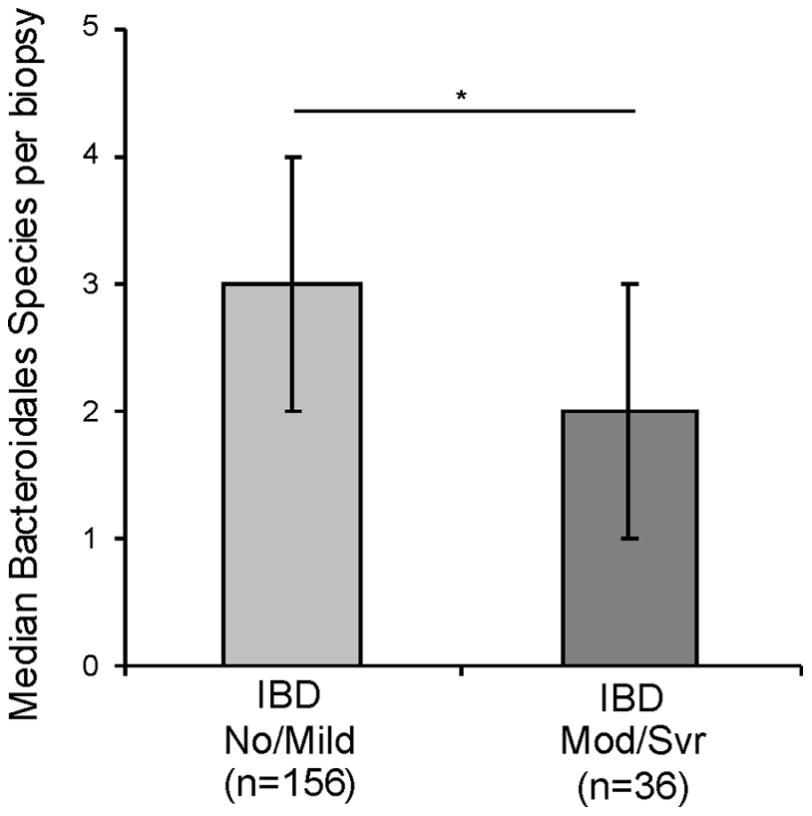
Bacteroidales species diversity by degree of inflammation. The median number of species per IBD biopsy detected from biopsies with no or mild inflammation (No/Mild) compared to IBD biopsies with moderate to severe inflammation (Mod/Svr). Error bars represent inter-quartile ranges. N equals the number of biopsies in each group. *P = 0.02.

In total, we identified between 0–9 different Bacteroidales species per biopsy (controls: 0–9, CD 0–7, UC 0–9) (Figure 2). Species diversity did not differ significantly in biopsies from IBD subjects compared to controls, nor did it differ significantly between IBD subtypes CD and UC (Table 2). Similarly, there were no significant differences in species diversity in biopsies from newly diagnosed subjects (Table S3). However, fewer unique Bacteroidales species were detected on moderate and severely inflamed IBD biopsies (median (IQR)  = 2 (1, 3) compared to mildly inflamed or uninflamed IBD tissue (median (IQR)  = 3 (2, 4); P = 0.03) (Figure 3, Table 2).

### Distribution of Bacteroidales species between IBD cohorts and between IBD and control biopsies

Species analyses yielded several statistically significant differences between CD, UC, and control groups. *P. distasonis* was present on (38%) of control biopsy samples compared to (14%) of CD biopsies (*P* = 0.003). *B. cellulosilyticus* was also present more often in control biopsies (8%) compared to CD biopsies (1%) (*P* = 0.04). *B. vulgatus* was present on significantly fewer biopsy samples from UC subjects (25%) compared to subjects with CD (53%, *P* = 0.03) and control subjects (51%, *P* = 0.05, Table 3).

**Table 3:** Comparison of Bacteroidales species, by disease status (IBD vs. controls) and study group (CD, UC, and controls).

	IBD vs Controls			Type of IBD			Secondary Hypothesis Tests		
Bacteroidales species^1^	IBD (n = 192)^2^	Control (n = 97)^2^	*P*	CD (n = 121)^2^	UC (n = 71)^2^	Control (n = 97)^2^	*P_CD vs UC_*	*P_CD vs Ctrl_*	*P_UC vs Ctrl_*
*P. distasonis*	32 (17%)	37 (38%)	**0.004**	17 (14%)	15 (21%)	37 (38%)	0.37	**0.003**	0.10
*B. fragilis*	84 (44%)	49 (51%)	0.47	54 (45%)	30 (42%)	49 (51%)	0.85	0.57	0.49
*B. thetaiotaomicron*	40 (21%)	29 (30%)	0.23	24 (20%)	16 (23%)	29 (30%)	0.76	0.24	0.45
*B. uniformis*	35 (18%)	31 (32%)	0.07	23 (19%)	12 (17%)	31 (32%)	0.80	0.14	0.10
*B. vulgatus*	82 (43%)	49 (51%)	0.43	64 (53%)	18 (25%)	49 (51%)	**0.03**	0.82	**0.05**
*B. ovatus*	51 (27%)	26 (27%)	0.97	33 (27%)	18 (25%)	26 (27%)	0.84	0.96	0.88
*B. caccae*	45 (23%)	15 (15%)	0.32	29 (24%)	16 (23%)	15 (15%)	0.88	0.32	0.46
*P. merdae*	26 (14%)	23 (24%)	0.15	15 (12%)	11 (15%)	23 (24%)	0.68	0.14	0.38
*B. cellulosilyticus*	4 (2%)	8 (8%)	0.08	1 (1%)	3 (4%)	8 (8%)	0.18	**0.04**	0.44
*B. dorei*	44 (23%)	16 (16%)	0.40	22 (18%)	22 (31%)	16 (16%)	0.22	0.83	0.16
*B. intestinalis*	2 (1%)	0 (0%)	--	1 (1%)	1 (1%)	0 (0%)	--	--	--
*B. stercoris*	18 (9%)	10 (10%)	0.85	11 (9%)	7 (10%)	10 (10%)	0.90	0.83	0.94
*B. eggerthii*	7 (4%)	4 (4%)	0.87	5 (4%)	2 (3%)	4 (4%)	0.67	1.00	0.67
*Dysgonomonas gadei*	0 (0%)	0 (0%)	--	0 (0%)	0 (0%)	0 (0%)	--	--	--
*B. xylanisolvens*	19 (10%)	5 (5%)	0.41	7 (6%)	12 (17%)	5 (5%)	0.19	0.10	0.15
*B. finegoldii*	24 (13%)	2 (2%)	0.08	15 (12%)	9 (13%)	2 (2%)	0.97	0.09	0.10
*Prevotella bivia*	0 (0%)	0 (0%)	--	0 (0%)	0 (0%)	0 (0%)	--	--	--
*B. acidifaciens*	0 (0%)	0 (0%)	--	0 (0%)	0 (0%)	0 (0%)	--	--	--

*P*-value from generalized estimating equation, controlling for within-subject correlation.

1. *B =  Bacteroides, P =  Parabacteroides*.

2. n is equivalent to the number of biopsies per cohort.

--: Prevalence too small for valid test of significance by GEE.

### Distribution of Bacteroidales species in newly diagnosed subjects compared to those with longstanding disease

Separate analyses of newly diagnosed IBD subjects were performed to avoid the potential effects that non-antibiotic medications may have on species distribution (Table S4). The distribution of *P. distasonis* had a similar trend to that of the entire cohort. *P. distasonis* remained significantly higher in controls compared to newly diagnosed patients with CD or UC in comparison to control subjects (*P* = 0.04 CD and *P* = 0.03 UC, Table S4). *B. finegoldii* was detected less often on control biopsies compared to UC biopsies (*P* = 0.01). However, in the cohort of new diagnoses, unique statistically significant differences emerged in the distribution of *B. thetaiotaomicron* and *B. caccae* that were not seen in the all inclusive cohort. *B. thetaiotaomicron* was more commonly present in UC compared to controls (*P* = 0.02) and compared to CD (*P* = 0.02). *B. caccae* was detected more often in newly diagnosed CD (45%) versus controls (15% *P* = 0.02) and UC versus control (*P* = 0.05) (Table S4, Figure 2). Some differences in species distribution between the newly diagnosed cohort were detected (Table 3, Table S4) suggesting duration of disease and/or that anti-inflammatory medications may modulate species distribution on the intestinal surface.

### Bacteroidales species from inflamed and uninflamed biopsies

In general, species detected on multiple biopsies within an individual showed a similar species distribution (Figure 2, Table 3 and S4). There were typically fewer species detected at inflamed areas compared to uninflamed areas within an individual (Figures 2 and 3). However, when comparing particular species distribution by degree of inflammation within our IBD cohorts, a few significant differences were observed. *P. distasonis* was detected on none of the moderate and severely inflamed biopsies from CD subjects and on 17% of biopsies with no or mild inflammation (*P* = 0.04). In newly diagnosed subjects, no statistical differences were found in species distribution between moderate and severely inflamed biopsies compared to mildly inflamed and uninflamed biopsies (data not shown). This was likely due to lower number of samples in each group providing inadequate power. We compared CD and UC biopsies with moderate and severe inflammation to those from control subjects in order to assess the effect of inflammation on species distribution. We detected decreased *B. vulgatus* in UC compared to control biopsies (*P* = 0.02, Table S5).

### Medication Effects

5-aminosalicylic acid, (5-ASA) Medications: 5-ASA has been proposed to affect bacterial growth and the amount of mucosal-associated bacteria. 12/39 of the CD subjects, 17/24 of the UC subjects, and 1/31 control subjects were treated with a 5-ASA medication at the time of sample collection. There was no statistically significant difference in total Bacteroidales concentration on biopsies from subjects receiving 5-ASAs. However, there was a significant decrease in Bacteroidales diversity (number of detected species) at inflamed sites in patients treated with 5-ASA, mean ± SD 2.1±1.9, compared to 4.2±2.6 in patients not on 5-ASA (*P* = 0.03). The only significant species difference associated with 5-ASA treatment was a decrease in *B. vulgatus* at moderate to severely inflamed sites (*P* = 0.01).

### Species diversity and total Bacteroidales per biopsy comparing medications other than 5-ASA

Previous groups have shown that anti-inflammatory medications used alone or in combination to treat IBD may have effects on the microbiota [32]. We compared species diversity (number of different Bacteroidales species detected by subject), and total Bacteroidales (cfu/biopsy) in IBD subjects compared to six control subjects who were not receiving medications. We compared medications alone and in combination (data not shown). There were no significant differences detected for either of these analyses, possibly due to the small number of subjects in each group.

### Bacteroidales growth on mucin

The Bacteroidales species detected in this study were predominant species adherent to the mucosal surface. The presence of Bacteroidales embedded in the mucosal surface is potentially associated with the ability of these bacteria to utilize mucin as a source of nutrients. We tested 373 of our isolates for their ability to grow on defined mucin plates where mucin is supplied as the only utilizable carbon source. Every strain tested grew on minimal mucin plates and these included: *B. caccae*, 22; *B. cellulocyliticus*,8; *B. dorei*, 27; *B. eggerthii*, 7; *B. finegoldii*,13; *B. fragilis*, 50; *B. intestinalis*, 3; *B. ovatus*, 38; *B. stercoris*, 13; *B. thetaiotaomicron*, 35; *B. uniformis*, 32; *B. vulgatus*, 46; *B. xylanisolvens*, 18; *P. distasonis*, 36; *P. merdae*, 24; and *Dysgonomonas gadeii* 1.

### Detection of *bft* [*B. fragilis* toxin gene]

**Figure 4:**
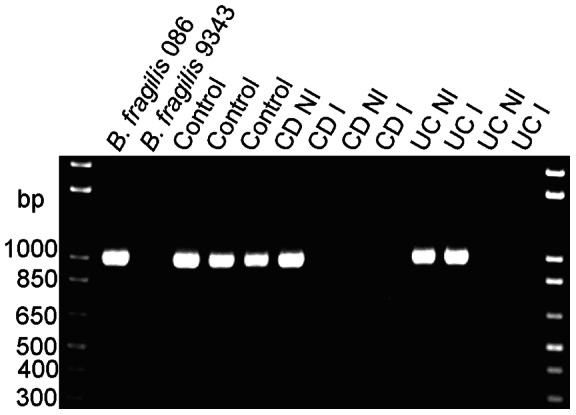
PCR analysis of *bft* from *B. fragilis* isolates. Lane one contains a *bft* containing strain (086 care of C. Sears, MD). Lane two contains a *B. fragilis* strain without *bft* (9343). Lane three contains an isolate from the terminal ileum (TI) of subject 34. Lanes four and five contain isolates from TI and ascending colon biopsies from control subject 43. Lane 6 contains an isolate from the non-inflamed terminal ileum of a subject 71 with CD. Lane 7 contains an isolate from an inflamed TI biopsy of subject 64 with CD. Lanes 8 and 9 contains isolates from non-inflamed rectal and inflamed ascending colon biopsies of a subject 74 with CD. Lanes 10 and 11 are isolates from non-inflamed TI and inflamed descending colon biopsies of a subject 39 with UC. Lanes 12 and 13 are isolates from an non-inflamed cecal biopsy and inflamed rectal biopsy from subject 62 with UC.

*bft* encodes for an enterotoxin that has been associated with colitis flares, colon cancer and diarrhea [33]. *B. fragilis* isolates cultured from biopsy tissue were screened by PCR for the presence of the *bft* gene. In total, 104 *B. fragilis* isolates were screened; 40 from control tissue, 21 from non-inflamed CD biopsies, and 20 from inflamed CD biopsies, 10 from non-inflamed UC biopsies, and 13 from inflamed UC biopsies. Of these 104 *B. fragilis* isolates, only six were *bft* positive (Figure 4, Table 4). Of these six *bft* positive isolates, three isolates were from biopsy tissue from control subjects (two biopsies were from the same control subject), one was from an uninflamed CD biopsy, and the remaining two were from uninflamed and inflamed biopsies from the same subject with UC. There were no statistically significant differences detected between UC and CD, between IBD and controls, or between strains from inflamed and uninflamed sites.

**Table 4:** Number of biopsies with *B. fragilis* where *bft* or PSA was present.

	Control	CD NI	CD I	UC NI	UC I
***bft*** ** positive ** ***B. fragilis^1^***	2/40	1/21	0/20	1/10	1/13
**PSA1 positive ** ***B. fragilis*** **^2^**	9/20	5/12	4/14	1/6	2/7

Abbreviations: CD =  Crohn Disease, UC =  Ulcerative Colitis, I =  Inflamed biopsy tissue, NI =  Non-Inflamed biopsy tissue where isolate originated.

Denominator represents all biopsies in a cohort where *B. fragilis* was detected and numerators indicate:

1. *bft* detected by PCR. (No statistically significant differences were detected between groups).

2. PSA1 detected by immunoblot. (No statistically significant differences were detected between groups).

### Analysis of PSA1 producing *B. fragilis* isolates

**Figure 5:**
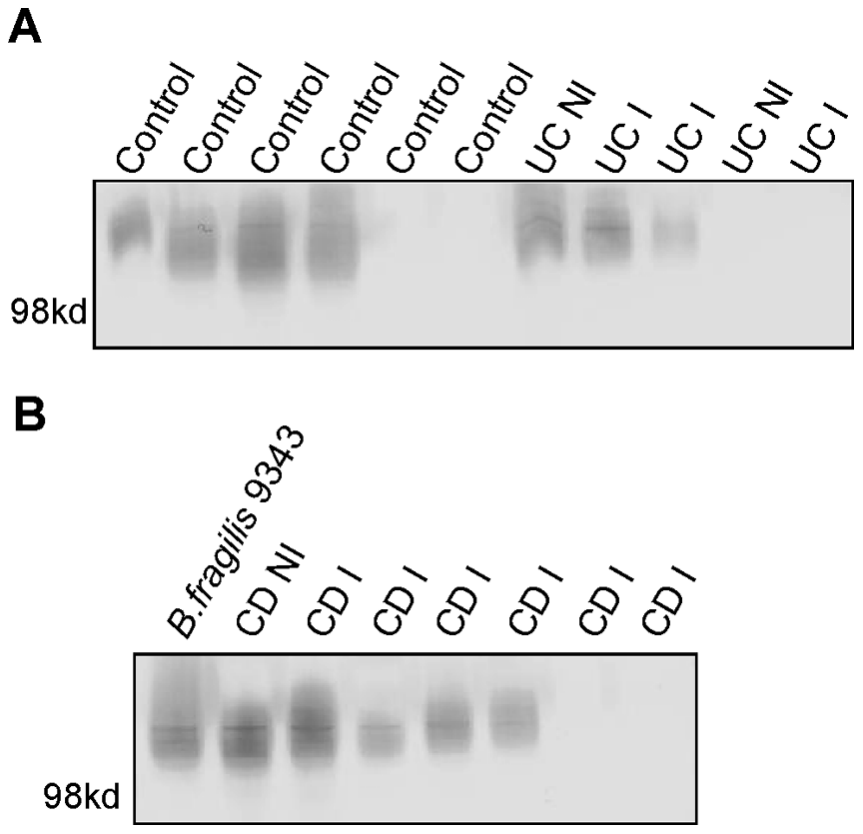
PSA analysis of *B. fragilis* isolates. A Western blot of PSA from *B. fragilis* isolates grown *in vitro* after isolation from control, CD and UC (inflamed (I) a non-inflamed (NI) biopsies. Panel A: Lanes 1–6 contain *B. fragilis* isolates from the biopsies control subjects (4, 6, 13, 34, 68, and 1). Lanes 7–8 contain isolates from non-inflamed and inflamed biopsies from subject 23 with UC. Lanes 9–11 contain isolates from inflamed and uninflamed biopsies from subjects with UC: 32, 29 and 77. Panel B: Lane 1 contains *B. fragilis* type strain 9343 as a positive control. Lanes 2 and 3 contain isolates from a non-inflamed and then an inflamed biopsy from subject 47 with CD. Lanes 4–8 contain *B. fragilis* isolates from inflamed biopsies of subjects 26, 56, 64, 14, and 85 with CD.

Each strain of *B. fragilis* synthesizes eight distinct capsular polysaccharides termed PSA-PSH [34]. For type strain NCTC 9343, the PSA polysaccharide [PSA1] is a zwitterionic molecule with immunomodulatory properties implicated in the amelioration of colitis [14]. As different strains of *B. fragilis* produce different PSA-types [15], we screened the *B. fragilis* isolates from our biopsy samples to determine if the presence of strains producing this molecule were present more frequently from healthy mucosal tissue. A monoclonal antibody specific to the NCTC 9343 immunomodulatory PSA type [PSA1] was used [15], [35], [36]. In total, 59 *B. fragilis* isolates were screened of which, 20 (34%) reacted with the monoclonal antibody to the PSA of the type strain. Despite the anti-inflammatory properties described for this molecule, PSA1-producing *B. fragilis* were not found more frequently at non-inflamed sites in subjects with IBD compared to uninflamed sites. PSA1 producing *B. fragilis* were present on seven of 14 inflamed biopsies from CD subjects and two of seven inflamed biopsies from UC subjects. In addition, there was not a statistically significant difference between the presence of mucosally adherent PSA1-producing *B. fragilis* from controls versus IBD samples (*P* = 0.73). There were also no statistically significant differences in PSA1 producing *B. fragilis* adherent to the mucosa at inflamed and uninflamed sites in CD (*P* = 0.68) or UC (*P* = 1.0). (*P* values were determined using a Fishers exact test to compare each group). A newly diagnosed subject with CD had a PSA1 positive *B. fragilis* isolate at both an inflamed and non-inflamed site. The *B. fragilis* strains from the same individual tended to all synthesize the immunomodulatory PSA or were all negative for this molecule regardless of inflamed or non-inflamed site (Table 4, Figure 5). However in one CD subject (56), two different *B. fragilis* strains were detected on the same inflamed sample, one producing the immunomodulatory PSA1 molecule, and one producing a different PSA type.

## Discussion

In this study, we quantified and identified to the species level mucosally adherent Bacteroidales species from the intestine of pediatric and young adult patients with IBD and controls. We focused on the Bacteroidales for several reasons. Bacteroidales occupy a vital niche at the intestinal mucosal surface where they have the potential to influence the host. Numerous studies have implicated Bacteroidales species in IBD [20], [37], [38]. In addition, Bacteroidales have been shown to modulate host immune and intestinal functions [12], [39]. No other study has classified Bacteroidales to the species level as comprehensively as this study and no other study used a culture based approach, which is the most accurate quantitative method. Within our cohort, we obtained biopsies from newly diagnosed IBD subjects, as well as subjects with longstanding disease. We collected detailed phenotypic information about our subjects, which allowed us to make observations regarding variables such as the severity of inflammation and potential medication effects. In addition, we created a valuable strain bank from which we could perform further phenotypic analyses of the isolated strains.

Several studies have reported increases or decreases in Bacteroidales in mucosal samples of IBD subjects compared to controls [8], [37], [40], [41], some also compare inflamed and non-inflamed sites [42]. No clear trend has emerged regarding total Bacteroidales in IBD. Unlike DNA-based approaches, our culture-based method guarantees precise measurements of live bacteria. We found no significant differences in total number of adherent Bacteroidales in IBD versus control or between CD and UC. There was large inter-individual variation in total Bacteroidales detected across our cohort- from a few subjects with no detected Bacteroidales to those with log 4 cfu/biopsy. The log scale variability in total Bacteroidales by IBD cohorts and between individuals is consistent with other studies which performed anaerobic culture of biopsies [43] [44]. This suggests there is not a “normal” amount of Bacteroidales present on all mucosal surfaces across subjects and may explain the varied conclusions of prior studies. There is a molecular basis for why the Bacteroidales are able to survive at inflamed sites. *B. thetaiotaomicron* has been shown to adapt to inflammation by turning on genes that metabolize host oxidative products [45] suggesting that at least some *Bacteroides* species may be better suited to survive in inflamed regions. As our study demonstrated numerous Bacteroidales species can survive at inflamed sites, this may be a shared feature of intestinal Bacteroidales species.

One of our significant findings was a decrease in Bacteroidales diversity, [number of different Bacteroides species per biopsy] at sites with increased inflammation in IBD subjects. Previous reports detected decreased bacterial diversity in IBD subjects compared to controls [41] but have not reported changes in diversity with increased inflammation. In general, we noted larger inter-individual variation and less intra-subject variation at the species level, similar to what other studies have shown with multiple colonic biopsies from one individual [46] [9] [6] and in multiple stool samples from the same individual [47].

There were no Bacteroidales species signatures for CD, UC or control biopsies. However, there were statistically significant differences in certain species between cohorts. *P. distasonis* was found more often in control subjects compared to CD subjects in both newly diagnosed subjects and those with longstanding disease. Its predominance at healthy and uninflamed sites suggests a preference for adherence to ligands at uninflamed host sites, or greater susceptibility to host inflammatory products. A recent study reported a decrease of all *Parabacteroides spp*. at inflamed compared to uninflamed sites [9], similar to our findings. Fecal samples from pediatric subjects with IBD noted a similar decreases in *Parabacteroides* in IBD subjects versus controls [48]. The only other two pediatric studies which examined the microbiota of intestinal biopsies found no difference in *P. distasonis* between IBD subjects and controls [22] [23]. Interestingly, a recent study demonstrated that oral administration of the membranous fraction of *P. distasonis* significantly reduced the severity of intestinal inflammation in murine models of colitis induced by dextran sulphate sodium (DSS) [49].

*B. thetaiotaomicron* and *B. fragilis* contain enzymes that will partially desulphate mucins [50] which may be a feature shared among all Bacteroidales. The ability to degrade mucin has been suggested as a way that members of the intestinal microbiota may contribute to chronic inflammation, by compromising the protection mucin provides the host epithelium [24], [51]. We examined the ability of the strains from this study to utilize mucin as a carbon source for growth. All 373 of the strains we examined, comprising 16 different Bacteroidales species, were able to grow on plates with mucin as the sole utilizable carbon source. Since this is a feature of all isolated Bacteroidales from both inflamed and uninflamed sites, it is likely that this common feature has a primary role in nutrient acquisition.

Some strains of *B. fragilis* produce an enterotoxin [*Bacteroides fragilis* toxin, *bft*], which is correlated with pediatric diarrheal disease, colitis flares, colon cancer, and exacerbation of colitis in mouse models [reviewed [33]]. There were no differences in presence of *bft* positive *B. fragilis* isolates between IBD subjects and controls or between inflamed an uninflamed tissue, although very few *bft* ^+^ *B. fragilis* isolates were identified in total. One prior study of an older cohort (mean age 45) detected *bft* ^+^ *B. fragilis* isolates more frequently from colonoscopic washings of active IBD patients than stool from control subjects [38]. Another study, found no significant differences in *bft* ^+^ and negative strains between active and inactive inflammation or between IBD subtypes similar to our findings [52]. We have previously reported *bft* carriage in fecal samples from adults as high as 57% of *B. fragilis* isolates [47], therefore, it is possible that adults harbor more *bft* ^+^ *B. fragilis* strains than children.

We investigated an additional strain specific feature of *B. fragilis*, the synthesis of an extensively studied immunomodulatory capsular polysaccharide, PSA. Previous work has shown that *B. fragilis* strain NCTC 9343 synthesizing the immunomodulatory PSA is able to protect mice from *Helicobacter hepaticus* inflammation [14]. Only 25% of *B. fragilis* strains previously analyzed were found to synthesize the PSA1 molecule [15]. Based on the anti-inflammatory activity of PSA1, it is possible that PSA1 producing *B. fragilis* are present more frequently at uninflamed sites or in biopsies from control sites versus inflamed sites of IBD patients. However, we found no significant differences between these cohorts. As the synthesis of PSA is under complex regulation [34] [53], it is possible that only a small population of *B. fragilis* with the ability to synthesize PSA1 were actually doing so.

5-aminoslicylic acid, 5-ASA has been used for decades to treat IBD. 5-ASAs affect the host mucosal immune response but also have been shown to affect distribution, growth, and transcriptional responses of gut bacteria [54]. Additionally, 5-ASAs have been shown to decrease total concentrations of mucosal associated bacteria on human colonic biopsies when compared to control biopsies or biopsies from subjects treated with azathioprine [32]. Our study showed 5-ASAs decreased mucosally adherent Bacteroidales diversity at sites of increased inflammation. As Bacteroidales and Firmicutes are the predominant bacterial phyla at the colonic mucosal surface, some of the therapeutic effect of 5-ASAs may be by decreasing the host's bacterial burden at mucosal sites. Further studies are need to investigate if Bacteroidales growth is affected by the presence of 5-ASA, if any species specific factors can explain the relative decrease in certain species at inflamed sites, and if the drug also reduces the mucosal burden of predominant Gram positive commensal species.

Further study of the mechanisms in which these dominant commensals interact with the inflamed and healthy host intestinal epithelium is warranted. Our valuable collection of strains from inflamed and healthy subjects will provide a resource for further study.

## Supporting Information

Table S1 **Biopsy distribution and location.**
(DOC)

Table S2 **Primers used in this study.**
(DOC)

Table S3 **Total Bacteroidales (cfu/biopsy) and number of different Bacteroidales species detected per biopsy by diagnoses and degree of inflammation in newly diagnosed subjects.**
(DOC)

Table S4 **Comparison of Bacteroidales species by disease status [IBD vs. controls] and study group [CD, UC, and controls] in biopsies from 17 newly diagnosed subjects.**
(DOC)

Table S5 **Comparison of Bacteroidales species distribution from biopsies with moderate or severe inflammation to biopsies from control subjects.**
(DOC)
