# Supplementary material for: Characterization of Adherent Bacteroidales from Intestinal Biopsies of Children and Young Adults with Inflammatory Bowel Disease
Source: PLoS One. 2013 Jun 11;8(6):e63686. doi: 10.1371/journal.pone.0063686 (PMC3679120; doi:10.1371/journal.pone.0063686)
Supplement: Table S1 — Biopsy distribution and location. (DOC) [file pone.0063686.s001.doc]

**Table S 1: Biopsy distribution and location.**

| Sample Location | Control  (*n*=31 subjects) | | Crohn’s Disease  (*n*=39 subjects) | | Ulcerative Colitis  (*n*=24 subjects) | |
| --- | --- | --- | --- | --- | --- | --- |
|  | Total Biopsies | Inflamed Biopsies | Total Biopsies | Inflamed Biopsies | Total Biopsies | Inflamed Biopsies |
| Ileum (*n*=74) | 29 | 0 | 32 | 20 | 13 | 0 |
| Cecum (*n*=16) | 2 | 0 | 10 | 5 | 4 | 3 |
| Ascending Colon (*n*=35) | 13 | 0 | 18 | 6 | 4 | 2 |
| Transverse Colon (*n*=53) | 24 | 0 | 17 | 3 | 12 | 8 |
| Descending Colon (*n*=41) | 11 | 0 | 16 | 6 | 14 | 8 |
| Sigmoid (*n*=39) | 15 | 0 | 14 | 2 | 10 | 7 |
| Rectum (*n*=45) | 17 | 0 | 14 | 3 | 14 | 12 |
| Total (*n*=303) | 111 | 0 | 121 | 45 | 71 | 40 |
