# Supplementary material for: Characterization of Adherent Bacteroidales from Intestinal Biopsies of Children and Young Adults with Inflammatory Bowel Disease
Source: PLoS One. 2013 Jun 11;8(6):e63686. doi: 10.1371/journal.pone.0063686 (PMC3679120; doi:10.1371/journal.pone.0063686)
Supplement: Table S2 — Primers used in this study. (DOC) [file pone.0063686.s002.doc]

**Table S2**: Primers used in this study.

| **Multiplex primers**^1^ | Sequence | Product Size (bp) |
| --- | --- | --- |
| Forward primer: all species | gtacacaccgcccgt |  |
| Reverse primers: |  |  |
| MP I |  |  |
| *B. thetaiotaomicron* | acctatgaaatcgttgttacg | 175 |
| *B. vulgatus* | ctttctctcttccgtatcattac | 237 |
| *B. dorei* | CCCCcgaatctcaaga | 366 |
| *B. fragilis* | gctaatcccccaatcatac | 412 |
| *B. caccae* | agatcgtttcccattgct | 521 |
| *B. ovatus* | atcaatattgcgtactcgaac | 639 |
| MP II |  |  |
| *P. distasonis* | gtatgacctcggtacgga | 175 |
| *P. merdae* | ctggtctttccagtttaagatc | 242 |
| *B. eggerthii* | atcccccttcgtttcc | 370 |
| *B. stercoris* | gagatagtggttagtaatagacggt | 450 |
| *B. uniformis* | ggtgtttcaattataaaacaataatc | 550 |
| *B. cellulosilyticus* | Tcatgtcaaagaacggttg | 624 |
| *B. intestinalis* | tgcgtttgcctcctact | 761 |
|  |  |  |
| ***B. fragilis bft* amplification^2^** | | |
| forward | cgcggcattattagctgcatgttcttaatg | 992 |
| reverse | gatacatcagctgggttgtagacatccca |  |
|  | | |
| ***B.vulgatus* and *B.dorei* differentiation primers** | | |
| Forward (shared) | tcgaaagtgtgggtatcaaacag |  |
| *B. dorei* reverse | actcgctattgctagctgaacc | 275 |
| *B. vulgatus* reverse | ttacggctttcaccgtaattcat | 259 |
|  | | |
| ***B. fragilis* and *B.finegoldii* differentiation primers** | | |
| Forward (shared) | gtacacaccgcccgt |  |
| *B. finegoldii* reverse | gtctacctttcgtctagactcg | 314 |
| *B. fragilis* reverse | gctaatcccccaatcatac | 412 |
|  | | |
| ***B.ovatus*, *B. xylanisolvens*, and *B.acidifaciens* differentiation primers** | | |
| *B. ovatus* forward | tatgaataaggatcggctaactcc | 540 |
| *B. ovatus* reverse | ccttacggctatactgtttccaata |  |
| *B. xylanisolvens* forward | tatgaataaggatcggctaactcc | 535 |
| *B. xylanisolvens* reverse | cggctaacctgtttccagat |  |
| *B. acidifaciens* forward 1 | CGAGGGGCAGCATGA | 659 |
| *B. acidifaciens* forward 2 | GGAGTTTGCTTGCAAACTTCC | 646 |
| *B. acidifaciens* reverse | CGGAGTTCTTCGTGATATCTAAGC |  |
|  | | |
| **16S ribosomal DNA amplification Primers** | | |
| 16S rRNA region 1 forward | GCAGCCGCGGTAATACG (521-537)^3^ | 286 |
| 16S rRNA region 1 reverse | GGACTACCAGGGTATCTAATCCTGT (806-782) |  |
| 16S rRNA region 2 forward | CCTGGCTCAGGATGAACG (18-35) | 790 |
| 16S rRNA region 2 reverse | GTGGACTACCAGGGTATCTAATCCT (808-784) |  |
| 16S rRNA region 3 forward | GCAGCCGCGGTAATACG (521-537) | 549 |
| 16S rRNA region 3 reverse | ACGAGCTGACGACAACCAT (1073-1055) |  |
| 16S rRNA region 4 forward | tgaaactcaaaggaattgacg (905-925) | 600 |
| 16S rRNA region 4 reverse | tacggctaccttgttacgactt (1513-1491) |  |

^1^Multiplex assays adapted from Liu et. al. FEMS Microbiol. Let. 2003. 222:9-16 and Zitomersky et.al. Infect. Immun. 2010. 79: 2012-2020.

^2^Primers described in Sears et. al. Infect. Immun. 2006. 74: 5595–5601

^3^ Primer locations in relation to *E. coli* 16S rRNA gene (bp location)
