# Supplementary material for: Characterization of Adherent Bacteroidales from Intestinal Biopsies of Children and Young Adults with Inflammatory Bowel Disease
Source: PLoS One. 2013 Jun 11;8(6):e63686. doi: 10.1371/journal.pone.0063686 (PMC3679120; doi:10.1371/journal.pone.0063686)
Supplement: Table S3 — Total Bacteroidales (cfu/biopsy) and number of different Bacteroidales species detected per biopsy by diagnoses and degree of inflammation in newly diagnosed subjects. (DOC) [file pone.0063686.s003.doc]

**Table S3. Total Bacteroidales (cfu/biopsy) and number of different Bacteroidales species detected per biopsy by diagnoses and degree of inflammation in newly diagnosed subjects**. *P*-value from generalized estimating equation, controlling for within-subject correlation.

|  | **Subjects** | **Biopsies** | **Total Bacteroidales (mean log cfu/biopsy ± SD)** | ***P*** | **Bacteroidales species identified per biopsy**  **Median (IQR)** | ***P*** |
| --- | --- | --- | --- | --- | --- | --- |
| **Group (new diagnoses only)** |  |  |  |  |  |  |
|  |  |  |  |  |  |  |
| IBD | 17 | 55 | 3.1 ± 1.0 | 0.74 | 3 (2, 5) | 0.55 |
| Control | 31 | 97 | 3.0 ± 0.9 |  | 3 (2, 4) |  |
|  |  |  |  |  |  |  |
| CD | 12 | 40 | 2.9 ± 1.1 | 0.11 | 3 (2, 4.5) | 0.15 |
| UC | 5 | 15 | 3.5 ± 0.6 |  | 4 (3, 6) |  |
|  |  |  |  |  |  |  |
| CD | 12 | 40 | 2.9 ± 1.1 | 0.76 | 3 (2, 4.5) | 0.93 |
| Control | 31 | 97 | 3.0 ± 0.9 |  | 3 (2, 4) |  |
|  |  |  |  |  |  |  |
| UC | 5 | 15 | 3.5 ± 0.6 | 0.10 | 4 (3, 6) | 0.10 |
| Control | 31 | 97 | 3.0 ± 0.9 |  | 3 (2, 4) |  |
|  |  |  |  |  |  |  |
| **IBD (18 subjects)** |  |  |  |  |  |  |
| Inflammation | 16 | 28 | 3.0 ± 1.0 | 0.25 | 3 (2, 6) | 0.64 |
| No Inflammation | 15 | 27 | 3.2 ± 1.0 |  | 3 (2, 5) |  |
|  |  |  |  |  |  |  |
| Moderate/severe inflammation | 9 | 13 | 2.6 ± 1.0 | 0.14 | 3 (2, 4) | 0.13 |
| No/mild Inflammation | 17 | 42 | 3.2 ± 1.0 |  | 3 (2, 6) |  |
|  |  |  |  |  |  |  |
| **CD (12 subjects)** |  |  |  |  |  |  |
| Inflammation | 11 | 21 | 2.8 ± 1.1 | 0.28 | 3 (2, 5) | 0.78 |
| No inflammation | 10 | 19 | 3.1 ± 1.1 |  | 3 (1.5, 4.5) |  |
|  |  |  |  |  |  |  |
| Moderate/severe inflammation | 7 | 10 | 2.3 ± 1.0 | 0.08 | 2.5 (1, 3) | 0.12 |
| No/mild Inflammation | 12 | 30 | 3.1 ± 1.1 |  | 3 (2, 5) |  |
|  |  |  |  |  |  |  |
| **UC (5 subjects)** |  |  |  |  |  |  |
| Inflammation | 5 | 7 | 3.6 ± 0.6 | 0.73 | 4 (3, 6) | 0.72 |
| No inflammation | 5 | 8 | 3.5 ± 0.7 |  | 4 (2.5, 6.5) |  |
|  |  |  |  |  |  |  |
| Moderate/severe inflammation | 2 | 3 | 3.6 ± 0.1 | 0.59 | 4 (3, 4) | 0.46 |
| No/mild Inflammation | 5 | 12 | 3.5±0.7 |  | 4 (3, 6) |  |
|  |  |  |  |  |  |  |
