# Supplementary material for: Characterization of Adherent Bacteroidales from Intestinal Biopsies of Children and Young Adults with Inflammatory Bowel Disease
Source: PLoS One. 2013 Jun 11;8(6):e63686. doi: 10.1371/journal.pone.0063686 (PMC3679120; doi:10.1371/journal.pone.0063686)
Supplement: Table S4 — Comparison of Bacteroidales species by disease status [IBD vs. controls] and study group [CD, UC, and controls] in biopsies from 17 newly diagnosed subjects. (DOC) [file pone.0063686.s004.doc]

| **Table S4. Comparison of Bacteroidales species by disease status (IBD vs. controls) and study group (CD, UC, and controls) in biopsies from 17 newly diagnosed subjects. *P*-value from generalized estimating equation, controlling for within-subject correlation.** | | | | | | | | | | | | | |
| --- | --- | --- | --- | --- | --- | --- | --- | --- | --- | --- | --- | --- | --- |
|  | **IBD vs Controls** | | |  | **Type of IBD** | | |  | | **Secondary Hypothesis Tests** | | | |
| **Bacteroidales species^1^** | **IBD**  **(n=55)** ^2^ | **Control (n=97)** | ***P*** |  | **CD**  **(n=40)** | **UC  (n=15)** ^4^ | **Control (n=97)** | |  | | ***P_CD vs UC_*** | ***P_CD vs Ctrl_*** | ***P_UC vs Ctrl_*** |
|  |  |  |  |  |  |  |  | |  | |  |  |  |
| *P. distasonis* | 6 (11%) | 37 (38%) | **0.01** |  | 5 (13%) | 1 | 37 (38%) | |  | | 0.55 | **0.04** | **0.03** |
| *B. fragilis* | 25 (45%) | 49 (51%) | 0.72 |  | 16 (40%) | 9 | 49 (51%) | |  | | 0.44 | 0.50 | 0.69 |
| *B. thetaiotaomicron* | 17 (31%) | 29 (30%) | 0.93 |  | 8 (20%) | 9 | 29 (30%) | |  | | **0.02** | 0.44 | **0.02** |
| *B. uniformis* | 16 (29%) | 31 (32%) | 0.80 |  | 9 (23%) | 7 | 31 (32%) | |  | | 0.24 | 0.47 | 0.42 |
| *B. vulgatus* | 32 (58%) | 49 (51%) | 0.58 |  | 23 (58%) | 9 | 49 (51%) | |  | | 0.92 | 0.65 | 0.69 |
| *B. ovatus* | 20 (36%) | 26 (27%) | 0.37 |  | 16 (40%) | 4 | 26 (27%) | |  | | 0.41 | 0.29 | 0.99 |
| *B. caccae* | 26 (47%) | 15 (15%) | **0.007** |  | 18 (45%) | 8 | 15 (15%) | |  | | 0.72 | **0.02** | **0.05** |
| *P. merdae* | 10 (18%) | 23 (24%) | 0.62 |  | 4 (10%) | 6 | 23 (24%) | |  | | 0.13 | 0.23 | 0.44 |
| *B .cellulosilyticus* | 1 ( 2%) | 8 ( 8%) | 0.16 |  | 1 ( 3%) | 0 | 8 ( 8%) | |  | | -- | -- | -- |
| *B. dorei* | 11 (20%) | 16 (16%) | 0.74 |  | 8 (20%) | 3 | 16 (16%) | |  | | 1.00 | 0.76 | 0.84 |
| *B. intestinalis* | 0 ( 0%) | 0 ( 0%) | -- |  | 0 ( 0%) | 0 | 0 ( 0%) | |  | | -- | -- | -- |
| *B. stercoris* | 11 (20%) | 10 (10%) | 0.21 |  | 9 (23%) | 2 | 10 (10%) | |  | | 0.44 | 0.17 | 0.70 |
| *B. eggerthii* | 2 ( 4%) | 4 ( 4%) | 0.91 |  | 2 ( 5%) | 0 | 4 ( 4%) | |  | | -- | -- | -- |
| *B. xylanisolvens* | 3 ( 5%) | 5 ( 5%) | 0.96 |  | 0 ( 0%) | 3 | 5 ( 5%) | |  | | -- | -- | -- |
| *B. finegoldii* | 12 (22%) | 2 ( 2%) | **0.03** |  | 6 (15%) | 6 | 2 ( 2%) | |  | | 0.27 | 0.10 | **0.01** |
| *D. gadei* | 0 ( 0%) | 0 ( 0%) | -- |  | 0 ( 0%) | 0 | 0 ( 0%) | |  | | -- | -- | -- |
| *P. bivia* | 0 ( 0%) | 0 ( 0%) | -- |  | 0 ( 0%) | 0 | 0 ( 0%) | |  | | -- | -- | -- |
| *O. splanchnicus* | 0 ( 0%) | 1 ( 1%) | -- |  | 0 ( 0%) | 0 | 1 ( 1%) | |  | | -- | -- | -- |

1*. B= Bacteroides, P= Parabacteroides* for *P.merdae* and *P.distasonis* and *Prevotella* for *P.bivia, D=Dysgonomonas*, O=*Odoribacter*

2. n is equivalent to the number of biopsies per cohort

3. -- Prevalence too small for valid test of significance by GEE.

4. Percentages is not reported for groups when n<30.
