# Supplementary material for: Characterization of Adherent Bacteroidales from Intestinal Biopsies of Children and Young Adults with Inflammatory Bowel Disease
Source: PLoS One. 2013 Jun 11;8(6):e63686. doi: 10.1371/journal.pone.0063686 (PMC3679120; doi:10.1371/journal.pone.0063686)
Supplement: Table S5 — Comparison of Bacteroidales species distribution from biopsies with moderate or severe inflammation to biopsies from control subjects. (DOC) [file pone.0063686.s005.doc]

| **Table S5. Comparison of Bacteroidales species distribution from biopsies with moderate or severe inflammation to biopsies from control subjects. *P*-value from generalized estimating equation, controlling for within-subject correlation.** | | | | | | | | | |
| --- | --- | --- | --- | --- | --- | --- | --- | --- | --- |
|  | **CD** |  | **UC** | **Control** |  | **CD vs UC** | **CD vs Ctrl** | **UC vs Ctrl** |  |
| **Bacteroidales species^1^** | **Mod/Severely Inflamed**  **(N=22)** *^2^* |  | **Mod/Severely Inflamed**  **(N=14)** | **(N=97)** |  | ***P_CD vs UC_*** | ***P_CD vs Ctrl_*** | ***P_UC vs Ctrl_*** |  |
|  |  |  |  |  |  |  |  |  |  |
| *P. distasonis* | 0 |  | 2 | 37 |  | -- | -- | -- |  |
| *B. fragilis* | 11 |  | 6 | 49 |  | 0.74 | 0.98 | 0.68 |  |
| *B. thetaiotaomicron* | 5 |  | 2 | 29 |  | 0.51 | 0.51 | 0.24 |  |
| *B. uniformis* | 2 |  | 2 | 31 |  | 0.70 | 0.06 | 0.35 |  |
| *B. vulgatus* | 10 |  | 1 | 49 |  | **0.04** | 0.73 | **0.02** |  |
| *B. ovatus* | 5 |  | 3 | 26 |  | 0.92 | 0.70 | 0.68 |  |
| *B. caccae* | 3 |  | 4 | 15 |  | 0.31 | 0.85 | 0.34 |  |
| *P. merdae* | 2 |  | 0 | 23 |  | -- | -- | -- |  |
| *B. cellulosilyticus* | 0 |  | 0 | 8 |  | -- | -- | -- |  |
| *B. dorei* | 2 |  | 3 | 16 |  | 0.36 | 0.41 | 0.73 |  |
| *B. intestinalis* | 1 |  | 0 | 0 |  | -- | -- | -- |  |
| *B. stercoris* | 2 |  | 1 | 10 |  | 0.84 | 0.87 | 0.73 |  |
| *B. eggerthii* | 1 |  | 0 | 4 |  | -- | -- | -- |  |
| *B. xylanisolvens* | 1 |  | 1 | 5 |  | 0.75 | 0.92 | 0.79 |  |
| *B. finegoldii* | 2 |  | 2 | 2 |  | 0.73 | 0.28 | 0.16 |  |
| *D. gadei* | 0 |  | 1 | 0 |  | -- | -- | -- |  |
| *P. bivia* | 0 |  | 1 | 0 |  | -- | -- | -- |  |
| *O. splanchnicus* | 0 |  | 0 | 1 |  | -- | -- | -- |  |

1*. B= Bacteroides, P= Parabacteroides* for *P.merdae* and *P.distasonis* and *Prevotella* for *P.bivia, D=Dysgonomonas*, O=*Odoribacter*

2. n is equivalent to the number of biopsies per cohort

3. -- Prevalence too small for valid test of significance by GEE.
